# Legs as linkages: an alternative paradigm for the role of tendons and isometric muscles in facilitating economical gait

**DOI:** 10.1242/jeb.243254

**Published:** 2022-03-08

**Authors:** James R. Usherwood

**Affiliations:** Structure and Motion Laboratory, The Royal Veterinary College, North Mymms, Hatfield, Hertfordshire AL9 7TA, UK

**Keywords:** Linkage, Work, Gait, Tendon, Isometric, Locomotion

## Abstract

Considerable attention has been given to the spring-like behaviour of stretching and recoiling tendons, and how this can reduce the work demanded from muscle for a given loss–return cycling of mechanical energy during high-speed locomotion. However, even completely isometric muscle–tendon units have the potential to act as tension struts, forming links in linkages that avoid the demand for mechanical work-cycling in the first place. Here, forelimb and hindlimb structures and geometries of quadrupeds are considered in terms of linkages that avoid mechanical work at the level of both the whole limb and the individual muscles. The scapula, isometric serratus muscles and forelimb can be viewed as a modified Roberts' straight-line mechanism that supports an approximately horizontal path of the body with vertically orientated forces, resulting in low work demand at the level of both limb and muscle. Modelled isometric triceps brachii inserting to the olecranon form part of a series of four-bar linkages (forelimb) and isometric biceps femoris cranial, rectus femoris and tensor fascia latae form part of a series of six-bar linkages (hindlimb), in both cases potentially resulting in straight-line horizontal motion, generating appropriate moments about shoulder and hip to maintain vertical ground reaction forces and again low mechanical work demand from the limb. Analysing part of the complexity of animal limb structure as linkages that avoid work at the level of both the whole limb and the supporting muscles suggests a new paradigm with which to appreciate the role of isometric muscle–tendon units and multiple muscle origins.

## Abstract (precis)

**Summary:** Muscles, tendons and bones form linkages that avoid much of the mechanical work demand of locomotion at the level of both the whole leg and the individual muscles.

## Introduction

**Fig. 1.**
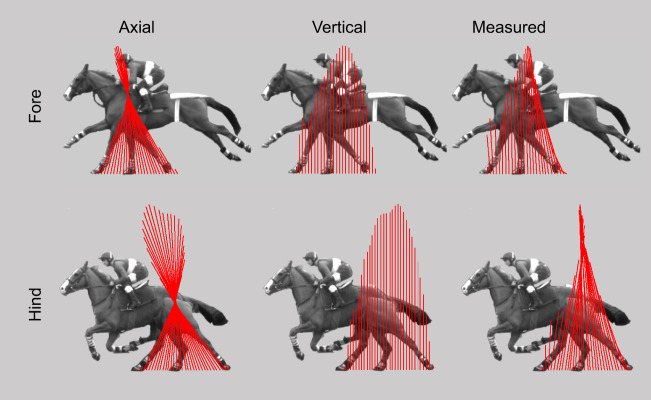
**Conterfactual and measured ground reaction force vectors (red lines) in a horse frame of reference for a forelimb and hindlimb stance in galloping.** In each case, vertical forces are those measured. The axial case shows the conventional approximation that minimises joint work, with force vectors aligned with a proximal joint (high scapular or hip). The vertical case aligns forces vertically, which, for a horizontal body path, results in zero power or work at the level of the limb (though high joint work). This is the approximation adopted in this paper. The measured force vectors demonstrate that forces are more vertical than purely axial, aligned well ahead of the proximal joint in early stance and behind in late stance. Data are from a study reported in Self Davies et al. (2019).

It has long been recognized that the fundamental mechanical work demands of steady, level locomotion would be low if weight could be supported with a horizontal centre of mass path and vertical forces from the supports. After all, this describes the situation with rolling wheels, sliding skates and skis. This has also been shown to be true for legged locomotion of quadrupeds (Usherwood, 2020) and, taking some account of the issues of pitching, in hopping and running bipeds (Alexander and Vernon, 1975; Usherwood and Hubel, 2012). However, the details of how vertical forces might actually be achieved with legs, without enormously costly simultaneous positive and negative powers from co-contracting muscles, is not immediately obvious. Vertical forces throughout stance would require large moments about the shoulder or hip – how might they be achieved without requiring muscle power as the leg rotates under the body? Further, horizontal forces are observed during steady, level, legged mammal locomotion, with a retarding force in early stance and a propelling horizontal force late in stance. For these reasons, it was assumed (e.g. Alexander, 1976, 1989) that the required mechanisms for the horizontal path, vertical force strategy do not exist in animals, and that, as a first approximation, forces should be considered as aligned with the hip or shoulder joints (Fig. 1) (here termed ‘leg-axial’ forces), minimizing the work costs of simultaneous positive/negative muscle power. This assumption has been adopted very widely and applied to considerations of work minimisation (Polet and Bertram, 2019), passive dynamics (Gan et al., 2016) and mechanical idealisations such as the spring-mass models of running (McMahon et al., 1987; Blickhan, 1989; McMahon and Cheng, 1990; Farley et al., 1993; Robilliard and Wilson, 2005; Geyer et al., 2006). The role of isometric muscles and tendon tension was viewed predominantly in the context of spring-like energy storage and recoil [Maynard Smith and Savage, 1955 (who cite Camp and Smith, 1942 and Simpson, 1951); Cavagna et al., 1964; Alexander and Bennet-Clark, 1977; Cuming et al., 1978; Alexander et al., 1982; Heglund et al., 1982; Alexander, 1984; Alexander and Dimery, 1985; Dimery et al., 1986; Ker et al., 1987; Roberts et al., 1997; Biewener et al., 1998; McGuigan and Wilson, 2003], reducing the mechanical work demanded from the muscles for a given limb work dissipation–generation cycle.

The leg-axial force assumption was a useful and necessary reduction at the time. However, it was also known at the outset (Jayes and Alexander, 1978) that this was not quite true: force vectors are actually orientated more vertically than leg-axially. Indeed, Jayes and Alexander (1978) report that force vectors approximately converge towards a point over double the hip height in sheep. This focus is even more extremely elevated in hindlimbs and forelimbs of galloping horses (2.5 or more; see Fig. 1 and Movie 1 presenting measurements from Self Davies et al., 2019), with important energetic implications: this geometry results in the horizontal work demanded from the limbs being reduced by 50–60% compared with limbs loaded with leg-axial forces of the same vertical forces. There is some evidence that more vertical than limb-axial forces is a general feature of quadrupedal gaits (see Usherwood and Granatosky, 2020). In this context, it appears at least equally valid to view the forces – especially during very high speed locomotion – as approximating vertical rather than leg-axial. If we take an alternative reductionist extreme, then, that the body passes horizontally over the feet (the feet pass horizontally under the body) and forces are entirely vertical, how might we interpret animal limb structure as mechanisms for achieving this situation passively, without enormous and costly simultaneous positive and negative muscle work? Might isometric muscles and tendons be viewed not as springs, reducing the muscle work demand for a given cycle of mechanical work, but as links enabling mechanisms that reduce the mechanical work demand in the first place?

## The cost of compression and the value of straight-line mechanisms

The geometry of a hip passing approximately horizontally over a foot requires the leg to compress (Farley et al., 1993). How could it be possible for energy not to be lost – whether in stretching a spring or extending a loaded muscle eccentrically? Equally, beyond midstance, the leg must extend; again, how can this be achievable without demanding positive power from a combination of elastic tendon recoil and concentric muscle contraction? Well, if the forces can be maintained vertically and the motions entirely horizontally, no net negative or positive power is required (this clearly contrasts with the leg-axially loaded case) – this geometry is how wheels, skates and skis can allow such economical weight-bearing translation. This paper introduces the principles of linkages in work-avoidance, and some methods for inferring the role of certain muscles in facilitating these linkages, by focusing on the low-work strategy of adopting approximately straight-line horizontal body motions with approximately vertical forces. It should be noted that this strategy is not the only one in which low mechanical work might be achieved with linkages (walking is not considered here). Further, it should be highlighted that linkage-based work avoidance is not the only strategy for avoiding some aspect of muscle work demand; elastic storage and recoil certainly provides some energetic benefits that may complement the linkage mechanisms. No attempt is made here to quantify the relative contributions of different work-reduction mechanisms; however, this would be an interesting and feasible question for future studies.

The aim of this paper is to demonstrate how gait with low mechanical work demand at the level of the limbs – in this case owing to approximately vertical forces and horizontal body path – might be achieved at the level of the muscles. In order for there to be a low mechanical work demand on the muscles, they must be isometric (constant length) whenever under tension. If they become slack, resisting no tension when the distance between origin and insertion becomes shorter for geometric reasons, no muscle work is required.

## The forelimb of cursorial mammals: a form of Roberts’ linkage

**Fig. 2.**
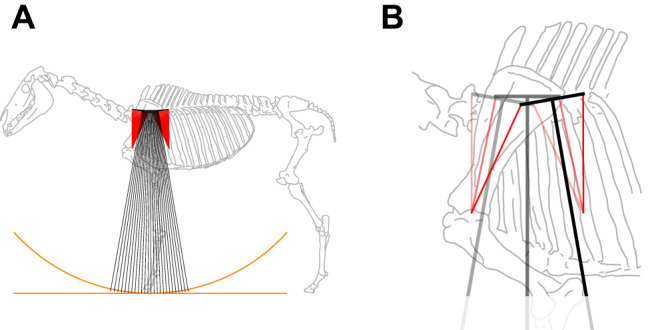
**Two views of the horse forelimb, serratus (muscle, red lines) and scapula modelled as a modified Roberts' straight-line mechanism.** (A) A full-body view indicating the foot path is more nearly horizontal (orange straight line) than arcing with radius of anatomical leg length (orange arc). (B) The close-up shows early (pale), mid (intermediate) and late (dark) snapshots. The serratus muscle ‘sling’ allows the ‘functional’ leg (including serratus attachment to the body) to shorten and extend over stance, while all elements (serratus, and the hoof-to-scapula ‘anatomical’ leg) remain idealised as isometric, facilitating economical near-straight-line motion of the centre of mass, low mechanical work demand at the level of the limb, and low muscle work requirements for support with isometric muscles. See Movie 2 and Supplementary Materials & Methods.

One approach to interpreting leg structure in the context of maintaining vertical forces and horizontal velocities is to search for anatomical analogies to classic ‘straight-line’ mechanisms described during the industrial revolution (Usherwood, 2020). One such mechanism is the Roberts' linkage: there appears to be a reasonably direct analogy with the structure and action of the typical quadrupedal mammal (and possibly more widespread) scapula supported by a ‘sling’ of serratus muscles (Fig. 2, Movie 2, Supplementary Materials & Methods for pattern for card model) (Gray, 1968; Goody, 1976; Carrier et al., 2006), suitable for resisting the tension forces imposed by body weight economically (Maynard Smith and Savage, 1955; Payne et al., 2005). It is conventionally viewed that the scapula/serratus arrangement extends stance length through an action that lifts the centre of rotation of the forelimb through combining a sliding translation with a rotation (English, 1978). But what limits this sliding? And if the sliding translation is beneficial, why is it not extended further? The Roberts' mechanism demonstrates how the foot is able to translate under the body – or the body over the foot – with broadly isometric tension and compression elements without demanding an arcing path about a pin-joint shoulder attachment to the body. The geometry of greyhounds and horses does not appear to allow a completely straight-line, optimal Roberts' linkage, but it does go some way to explaining the mechanism underlying the sliding action, resulting in vertical forces, horizontal velocities and low work demand of weight support, achieved with isometric and therefore low work demand at the level of the muscle.

Timings of muscle activity measured through electromyography (EMG) of dog muscles (Deban et al., 2012; see also Jenkins and Weijs, 1979 for Virginia opossum) broadly support the proposed action of the serratus muscles: activity is high in serratus ventralis cervicis (towards the head) in very early stance, and in serratus ventralis thoracis (behind the scapula) later on in stance. This is consistent with the Roberts' mechanism model; however, it is not altogether surprising that the forward tension elements support a greater load in early stance, and the rearward elements more in later stance. Therefore, it may be better to interpret the scapula/serratus arrangement not as for extending stance with sliding; rather, it allows functional leg shortening/lengthening over stance (Fig. 2) without tissue deflection, thereby avoiding unnecessary limb and muscle work.

The near-straight-line Roberts' reduction of the serratus and scapula also ties in with the observed moments and near-vertical orientation of ground reaction force vectors. In early stance, force vectors are orientated ahead of the scapula. At this point, the more cranial serratus (serratus ventralis cervicis) approximates vertical (Fig. 2) and the more caudal serratus (serratus ventralis thoracis) is inclined more horizontally; tension along both muscles would provide the moment acting to retract the leg (pushing the force vector from leg-axial towards vertical). At midstance, the cranial and caudal serratus muscles are balanced, generating no moment about the scapula and a vertical force. In late stance, the situation in early stance is reversed, with the cranial muscle inclined and caudal muscle vertical, generating a protracting moment about the scapula, again consistent with re-aligning a leg-axial ground reaction force towards vertical.

The reduction of the scapula and serratus muscles as a modified Roberts' linkage is clearly extreme: only two serratus muscles are considered, whereas in reality there is a fan of multiple muscle elements. And the muscles are only considered in their isometric, weight-supporting action here; if this were their only function, completely passive tendons would be less metabolically costly, and loading metabolically costly serratus muscle could – on an evolutionary timescale – be entirely avoided. Presumably, these muscles also have some role in performing work and power, or in supporting weight under a range of conditions that could not be achieved with tendons acting as obligate tension struts.

Scapula motions are clearly not the only joint motions during stance of the forelimb. The very distal joints deflect over large angles during galloping, associated with stretching and recoil of largely elastic tendons (Alexander and Dimery, 1985; Dimery et al., 1986; McGuigan and Wilson, 2003). However, the deflections of the distal joints and elastic recoil are not the focus of this study; instead, we continue with the question of whether linkages might enable low-work weight support during translation, and move on to consider the elbow joint.

Let us consider what geometries might allow muscles to act as isometric tension struts while resulting in horizontal straight-line motion of the hips or shoulders, and thus vertical-force weight support. Such mechanisms need not be working in isolation (the scapula/serratus Roberts' linkage and more elastic, distal joints may also influence joint geometry). However, if we assume that perfectly straight-line motion might be achieved with a single mechanism, we can use the required changes in joint geometry to search for potential lines of action of isometric muscle–tendon units. The following figures are intentionally somewhat ‘bare-boned’ as the approach for the rest of the paper is not in starting off with the muscles in order to calculate the actions; rather, it is to: use bone geometries and make assumptions concerning the actions (low mechanical power through horizontal motions and vertical forces); then calculate the rays of tension elements that would support these actions isometrically; and only then to suggest which muscles might fulfil the role of the rays.

## The forelimb as a series of four-bar linkages

**Fig. 3.**
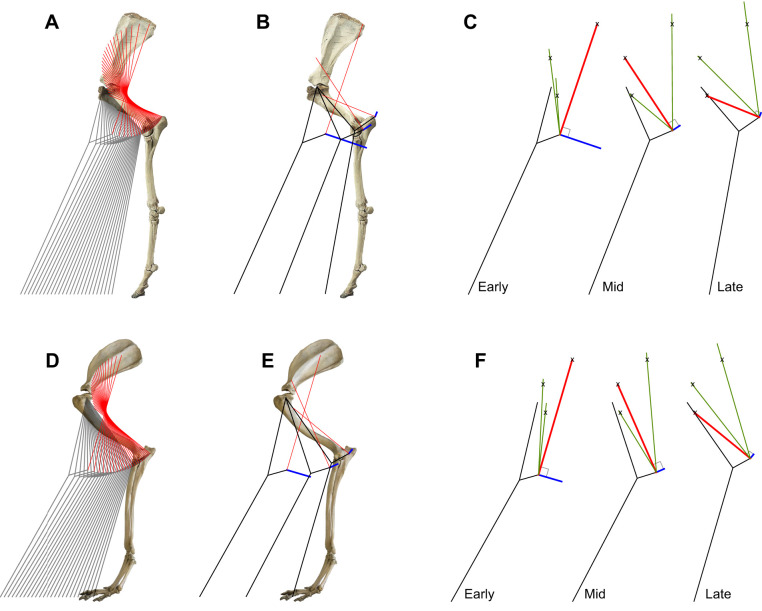
**The forelimb and rays (red lines) forming a series of four-bar linkages supporting straight line motion in two species.** (A–C) Horse; (D–F) dog. Calculated rays (red) approximate the triceps brachii, and are perpendicular to the instantaneous olecranon path (blue lines); the muscle elements would be isometric, and therefore able to resist tension with zero mechanical power. Ray lengths shown are arbitrary, but are sufficiently long so that each ray is longest (loses all slack; is under tension) when, and only when, perpendicular to the olecranon path. At all other times, the full-length ray (green lines, C,F) exceeds the insertion to putative origins (denoted by ‘x’), meaning that any changes in muscle length can occur when the muscle is slack and unloaded. In early stance, the predicted isometric muscle ray originates high on the scapula, approximating the long head of the triceps. When the foot is directly under the shoulder later in stance, a muscle element attaching to the humerus would lock the elbow joint, at this point resulting in the exactly vertical instant of a vault (exactly horizontal motion of the foot under the body). This would be achieved with the tension in the lateral head of the triceps. No attempt is made here to combine the Roberts' linkage (Fig. 2) with these series of four-bar linkages; either or both may be more or less effective in different animals or at different gaits. See Movie 3 and Supplementary Materials & Methods for physical model templates.

In the case of the forelimb, the olecranon or ‘point of elbow’ is a prominent site of muscle insertion. If straight-line motion at the shoulder were to be achieved, and the leg treated as consisting of two rigid boney parts (the humerus, and all the elements below the elbow, treated as two solid units), then the path throughout stance – and the direction of the velocity vector – of the olecranon can be determined given suitable bone geometry (Fig. 3, Movie 3, from lateral photographs of mounted horse and dog skeletons). Lines of muscle tension perpendicular to the instantaneous olecranon velocity are necessarily – at that instant – isometric.

If any single element of this fan were to oppose the moments about the elbow over the whole of stance it would deflect under load, performing mechanical power. This would appear as a net horizontal force, and mechanical work also at the level of the whole limb. However, if each element of the fan of muscles were only under tension at the instant they were isometric (when perpendicular to the olecranon path), there would be no mechanical work at the level of the muscle, and the net forces would be perfectly vertical, with zero work at the level of the limb. This serial loading of isometric elements of muscle could be appropriately achieved through simple geometry: each ray is at its longest at the instant it is perpendicular to the olecranon path (Fig. 3C,F, simply demonstrated with card-and-string models; templates in Supplementary Materials & Methods). Assuming muscle elements do act as purely tension struts, they are or become slack and impose no force (and so no mechanical power) at any instant their length determined from their origin-to-insertion geometry is shorter than their longest ‘slack-taken-up’ length.

The calculated fan of potential isometric tension struts (Fig. 3) broadly matches those of the triceps, which originates along the scapula and proximal humerus, and inserts at the olecranon. The predicted pattern of loading and strain predicted with this isometric strut and linkage analysis broadly agree with EMG measurements of cantering horse triceps (Hodson-Tole, 2006; Harrison et al., 2012) and rats (Schumann et al., 2002), and EMG and sonomicrometry measurements of muscle strain for galloping in goats (Carroll and Biewener, 2009): the long head (originating on from the caudal margin of the scapula) is active in stance well before the lateral head (originating from the humerus); and the long head is isometric (even if briefly) in early stance, before shortening, whereas the lateral head stretches in early stance, is then isometric, and subsequently shortens over late stance.

Although the potential for four-bar linkages to result in approximate straight-line motion and low-power weight support has been identified previously (Usherwood, 2020), no specific bone and muscle geometry has been successfully identified that achieves this. The analysis here demonstrates how a series of four-bar linkages might be automatically engaged through simple geometric means, resulting in straight-line, low-work motion. Note that, in contrast with the previous consideration of a single four-bar linkage tuned to achieve very nearly straight-line motion, the approach here (and below, for the hindlimb) calculates the series of linkages – each with a different muscle ray as one of the links – that achieves exactly straight-line motion, the joints of each linkage undergoing infinitesimally small changes in angle over the course of its engagement. The isometric but active nature of the triceps during weight support has previously been alluded to through geometric analysis and EMG recordings (Goslow et al., 1981); however, at that time, the functional implication of such loading was constrained to an assumption of benefit from elastic loading and recoil. The paradigm proposed here is that the broadly isometric loading of the triceps contributes to near-straight-line, vertical-force, low-power weight support. It also begins to point to how animals might benefit from the complexity of multiple muscle heads in the triceps (in addition to allowing different fibre types for different loading environments; Ryan et al., 1992): although each element of the muscle may have the same ‘action’ about a joint, having different origins allows the different elements to support body weight while isometric at different stages of stance.

## The hindlimb: a series of Watt's six-bar linkages

**Fig. 4.**
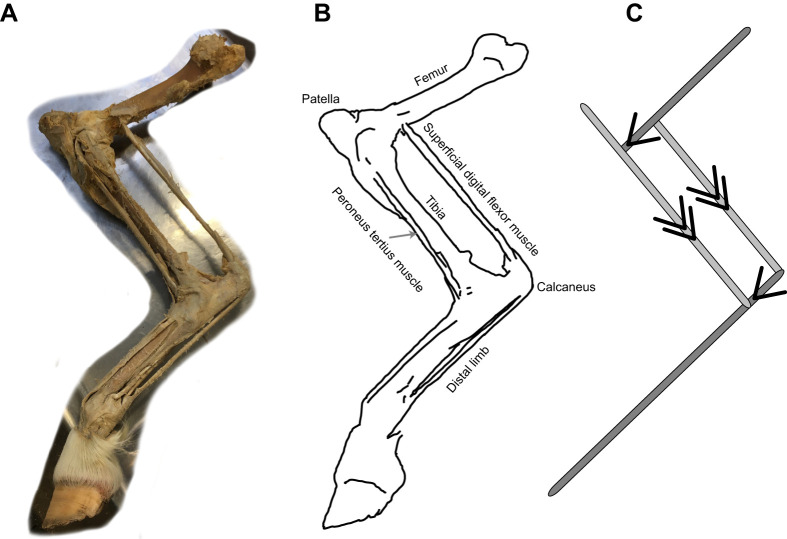
**A distal horse hindlimb displaying the ‘reciprocal apparatus’, parallelogram four-bar linkage or ‘pantograph’ mechanism.** (A) A dissected, formalin fixed preparation used in teaching. (B) An annotated sketch. (C) A reduction of the linkage highlighting the parallelogram property: the pair of lines with single-headed arrows (or feathers) are kept parallel; lines with double-headed arrows are kept parallel with each other. In the horse, the superficial digital flexor and peroneus tertius muscles have minimal in-series muscle, and the parallelogram four-bar mechanism is near-obligate; in dogs and other quadrupeds this is less extreme, but femur and distal limb section orientation remain closely coupled. This four-bar linkage is treated as a consistent distal element of a Watt's six-bar linkage formed from connected distal and proximal four-bars (Fig. 5).

In attempting to account for the perceived absence of suitable linkages in animal limbs, Alexander and Vernon (1975) noted that ‘an animal could be designed to walk or hop using vertical forces with quite low energy consumption, but most of its leg muscles would have to be two-joint muscles and it seems unlikely that it would be well adapted for any but the most stereotyped movement’. This assertion provides a useful starting point when looking for candidate linkages: look for cases where aspects of leg mechanism are very highly stereotyped. The ‘reciprocal apparatus’ of horses would therefore present an excellent place to start (Fig. 4). In the horse, the parallelogram formed by (1) the tibia opposite (3) the superficial digital flexor muscle SDF (or, if this would be loaded under compression, the tendinous tertius muscle, which is also parallel to the tibia but on the cranial side), and (2) the knee–SDF origin distance on the femur, opposite (4) the ankle–SDF insertion point on the calcaneus, forms a near-obligate four-bar linkage. The horse SDF and peroneus tertius have very little in the way of in-series muscle (Fig. 4 shows a formalin fixed leg prepared for teaching), meaning that, neglecting stretch of the SDF tendon between the femur and calcaneus, the femur and distal limb are coupled to be parallel. The implications of this four-bar linkage has been thoroughly discussed in terms of contributing to a passive, locking ‘stay’ mechanism (e.g. Hildebrand, 1987), allowing the horse to lock the leg in weight-bearing extension during standing, reducing the isometric, zero-work tension imposed on muscle. However, the linkage is also present (to a greater or lesser extent) in many mammals, most of which do not have the specific ‘stay’ adaptations. Might this linkage facilitate economical weight support throughout a hindlimb stance?

**Fig. 5.**
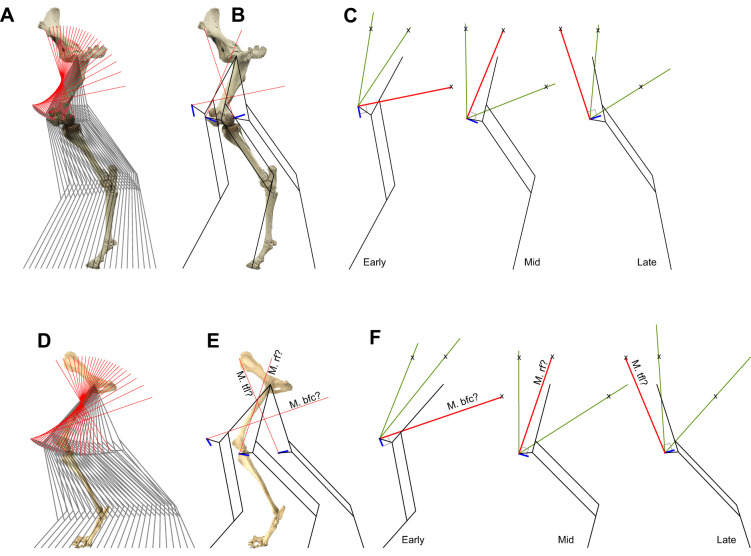
**Hindlimbs modelled as Watt's six-bar linkages (connected proximal and distal four-bar linkages) with a fan of putative proximal muscles resulting in straight-line motion.** (A–C) Horse; (D–F) dog. Each of the fan of elements is longest, loses all slack and provides tension only when perpendicular to the instantaneous path of the patella (blue lines, B,C,E,F); straight-line, work-avoiding motion is achieved without simultaneous positive and negative muscle power. At all other times, the full-length rays (green lines, C,F) exceed the patella insertion to putative origins (denoted by ‘x’) distance, meaning that any changes in muscle length can occur when the muscle is slack and unloaded. In early stance, a tension strut crossing the femur (matching the biceps femoris; Fig. 6A) produces a crossed quadrilateral for the proximal four-bar, and couples limb retraction with limb shortening, with appropriate moments about the hip resulting in vertical forces applied at the foot. In mid stance, the rectus femoris would allow approximate vaulting, with forces vertical and the foot directly beneath the hip. In late stance, the putative muscle (matching the tensor fasciae latae) loses slack and comes under tension, producing an uncrossed quadrilateral for the proximal four-bar, coupling retraction with limb extension, reversing the sense of moments about the hip, maintaining the vertical forces at the foot. See Movie 4 and Supplementary Materials & Methods for physical model templates. bfc, biceps femoris cranial; rf, rectus femoris; tfl, tensor fasciae latae.

**Fig. 6.**
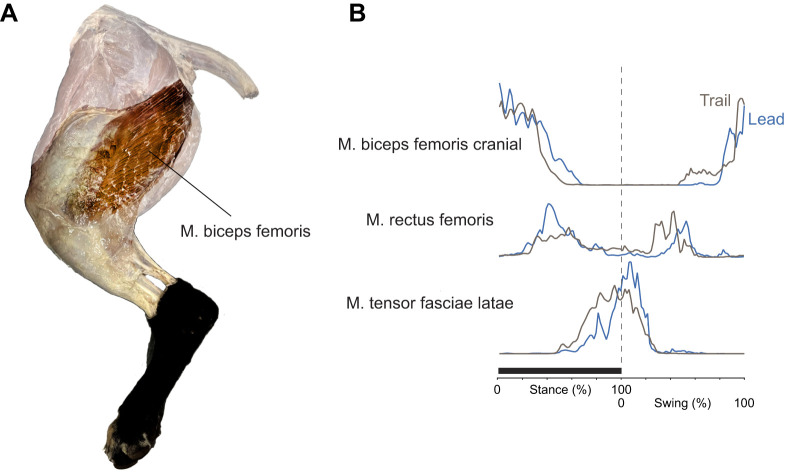
**Dog hindlimb anatomy and electromyography (EMG).** (A) The formalin fixed teaching specimen is presented, through dissection and digital manipulation, to highlight the biceps femoris muscle aligned from a broad origin behind the hip across the femur towards the patella insertion. (B) EMG observations reported for galloping dogs (Deban et al., 2012) agree with the timing of passive, geometric tension-loading of the putative muscles (Fig. 5): in early stance, the biceps femoris cranial is active; in mid stance, the rectus femoris is active; and in late stance, the tensor fasciae latae is active.

Tension elements attaching to the patella may result in such a linkage: multiple muscles insert on the patella, with origins ranging the full length of the pelvis. In order to analyse this possibility, the motions of the knee are calculated assuming exactly straight-line motion of the hip over level ground, or of the foot under the body. Further assuming the distal four-bar linkage to consist of perfectly isometric struts connected with free pin-joints (and limiting the analysis to the parasagittal plane) makes the path of the system explicit. As discussed for the triceps and olecranon, a muscle tension applied perpendicular to the path of the patella insertion would not change the length of the muscle – it would be isometric muscle, and no mechanical work need be performed by the muscle. The resulting kinematics are shown for horse and dog hindlimb (Fig. 5, Movie 4), with bone geometries from lateral-view photographs of mounted skeletons.

Horizontal straight-line motion is achievable with a fan of isometric tension elements inserting on to the patella. The origin of each ray of the fan displayed is not determined *a priori* by any anatomical points. The derived fan of tension elements are of arbitrary length, but it is important to note here (as also in the olecranon insertion description for the forelimb) that these lengths are sufficiently great for the fan elements, in a body frame of reference, to cross. This is a necessary condition for each tension element inserting at the patella to be at maximal length at – and only at – the instant at which they are perpendicular to the patella path. At all other positions through stance (whenever the tension line is not perpendicular to the patella path) the patella insertion is closer to the origin; the element becomes slack. Note that this does not require that the elements actually overlap (see Movie 4 and string-and-card model templates in Supplementary Materials & Methods).

This analysis effectively describes the hindlimb at each instant as a Watt's six-bar linkage; that is, two connected four-bar linkages. The distal parallelogram four-bar linkage (Fig. 4) couples the femur angle with the distal limb angle. It may be considered a ‘pantograph’ (Alexander, 1984), amplifying the action of the proximal linkage. The proximal four-bar linkage consists of the femur and patella, and of changing isometric tension element between the knee and near-pelvis origin, with the hip to near-pelvis origin also varying through stance and forming the fourth link. Which proximal four-bar linkage is engaged changes automatically through stance, as each of the tension elements inserting to the patella loses all slack or ‘takes the strain’. In early stance, the patella-inserted tension element crosses the femur, and the origin is some way behind the hip. This cross-over (‘self-intersecting’, ‘bowtie’ or ‘butterfly’) quadrilateral results in: (1) a coupling of limb retraction with limb shortening, as required for straight-line motion; and (2) applies a moment about the hip owing to the tension element origin behind the hip, matching (3) an external moment owing to the vertical ground reaction force on the foot placed in front of the hip. A candidate muscle for this link would be the cranial head of the biceps femoris, which originates on or close to the ischiatic tuberosity (point of the buttock). The biceps femoris muscle in dogs (Fig. 6A) and horses (and perhaps quite generally among quadrupedal mammals, but notably not humans) is fan-like and crosses the femur, broadly consistent with the fan of putative zero-work tension rays predicted from the linkage geometry.

In late stance, the predicted isometric tension element does not cross the femur; at this point, the simple (uncrossed) quadrilateral (1) couples limb retraction with limb extension, as required for straight-line motion once the foot has passed under the hip, and (2) applies a moment about the hip owing to tension elements connected to the pelvis ahead of the hip, matching (3) an external moment owing to the vertical ground reaction force on the foot placed behind of the hip. Candidate muscles for this link include the rectus femoris component of the quadriceps femoris muscle (with the origin closer to the hip), then part of the tensor fascia latae (origin at the most cranial part of the pelvis, the coxal tuber of ilium or ‘point of haunch’).

The order of tensioning predicted here matches EMG activity measurements for dogs across a range of gaits (Deban et al., 2012): early stance m. biceps femoris cranial, mid stance m. rectus femoris and late stance m. tensor fasciae latae (Fig. 6B). Similar activity phasing is reported for biceps femoris and tensor fasciae latae in horse at trot and canter (Tokuriki and Aoki, 1995). Further, EMG and sonomicrometry strain measurements in galloping goats (Gillis et al., 2005) and rats (Gillis and Biewener, 2001) show the biceps femoris is active (presumably under tension) but approximately isometric during the first half of stance, consistent with the role of the biceps as the tension strut forming the cross-over link that passively couples leg retraction with leg shortening. This function also provides some account for why a muscle whose concentric contraction would clearly propel and add energy to the system (and presumably does so during acceleration or climbing) should be active in the first half of stance during steady, level locomotion, when energy is in reality (moving away from the zero-work model idealization) being somewhat dissipated by the limb.

The principle of crossed and uncrossed four-bar linkages enabling energetically beneficial horizontal translation with vertical forces, with isometric muscles therefore demanding little work at the level of the muscles, was effectively alluded to as an ‘obvious’ theoretical possibility by Alexander (1976) in the context of bipedal walking; however, it was dismissed in this context, and does not appear to have been adopted subsequently in consideration of quadrupedal gaits.

## What are tendons and isometric muscles in animal legs ‘for’?

Tendon strains – whether positive or negative – alter the loading environment of connected muscles with a range of potential consequences for a given gross kinematic demand. Perhaps most obviously, tendons that stretch under load when work is being lost by the limb, and that recoil when the limb contributes mechanical work, can be viewed as elastic springs: the mechanical work demanded from the muscle for the action can be reduced [see Introduction; also (largely from Biewener, 2003); Biewener and Baudinette, 1995 (wallabies); Ker et al., 1987 (humans), Roberts et al., 1997 (turkeys); and Cavagna et al., 1977 (dogs and other trotting animals)]. This might be termed the ‘spring paradigm’. Appropriately tuned tendons can also improve the loading environment for muscle for a given positive work demand, in some ways reducing the metabolic cost of a unit of muscle work (modelled in Lichtwark and Wilson, 2007; questioned by Holt et al., 2014) and decreasing the instantaneous muscle power required for a high-power action (Aerts, 1998); and tendon deflection can alter the timing of muscle energy dissipation, reducing the instantaneous peak demands and again improving the loading environment experienced by the muscle, for instance when withstanding a fall (Konow and Roberts, 2015).

The question arises: which is the more important role of tendon in steady, level locomotion? The ‘spring paradigm’, with elastic storage and recoil from the tendon reducing the muscle work demand for a given mechanical energy cycle, or the ‘linkage paradigm’, where energy fluctuations are avoided? A quantitative answer to this is beyond the scope of this paper; it is not a trivial question to approach, and is likely to depend on both species and gait. Assessing the proportion of muscle work reduction that is due to elastic mechanisms requires either an accurate measure of both mechanical and metabolic works and a reliable value for muscle efficiency (see Cavagna et al., 1964), or measurement and/or modelling of the majority of elastic structures within the leg. Both methods have their challenges, and joint-by-joint analyses are clearly problematic once two-joint muscles need to be considered (see Gregersen et al., 1998).

An upper bound on the work avoided through linkages can be found by comparing the measured mechanical work with that which would be demanded without linkages. Research in the galloping horse (Self Davies et al., 2019) suggests this to be approximately 50% work avoidance, with similar values for goat and dog (Jayes and Alexander, 1978), assuming that limb-axial force vectors with empirical vertical forces provide a suitable ‘no-linkage’ counterfactual for comparison. However, this is insufficient for demonstrating that passive linkages actually pass these savings down to the level of the muscle. Although a range of feasible linkages has been described here, it is possible that work avoidance observed at the level of the whole limb is actually the result of costly simultaneous positive and negative muscle powers. To complicate matters further, the spring and linkage paradigms may well be complementary, but non-negligible deflections of the tension structures quickly invalidates the assumptions described here, allowing linkage geometries to be simply approximated.

And so it would certainly be premature to suggest that the current spring paradigm of tendon function in running animals should be discarded. But the linkage paradigm does begin to bring together observations of gross anatomy, limb mechanics and muscle activation in fast quadrupedal mammals by relating the conditions for low mechanical work at the level of the legs to the geometry of low work demand at the level of the muscles.

## Supplementary Material

Supplementary information
