# Supplementary material for: Legs as linkages: an alternative paradigm for the role of tendons and isometric muscles in facilitating economical gait
Source: J Exp Biol. 2022 Mar 8;225(Suppl 1):jeb243254. doi: 10.1242/jeb.243254 (PMC8987730; doi:10.1242/jeb.243254)
Supplement: Supplementary information [file jexbio-225-243254-s1.pdf]

Supplementary Materials and Methods.

Instructions

The outlines are derived from Figures 2, 3 and 5. Print templates on to thick card (or paper, then spray mount to card). Note that the 'parts' pages have duplicate parts to limit waste. Cut around grey lines. Red circles show joints; blue circles points of muscle attachment; black circles along the horizontal lines key points to facilitate tuning of muscle lengths. Red and blue circles should be punched or drilled (approx. 2.5mm appropriate for wool muscles and 4.5mm x 8mm split- pin brad fasteners for joints). When assembling, work from the distal (hoof, foot) end towards the body. For muscles with origins on the 'body', achieving appropriate 'muscle' (thread, string or wool) tension can be achieved by passing the muscle through the punched hole and wrapping around a matchstick, and held at that length by taping the matchstick/muscle to the back surface of the 'body'. Before taping, twist the matchstick to shorten the muscle such that the foot 'joint' hole aligns with the black circle on the 'body' dashed horizontal line appropriate for each leg angle (refer to figures).

A card link is used to represent the action of the SDF and/or peroneus tertius of the hindlimb; the combined action of these tendons is produced by a strut that opposes both tension and compression, with free pin joints at each end. It may be convenient to order the hindlimb segments with: SDF link closest to 'body' base; femur and distal segments one layer above; and tibia with multiple muscle attachments at the patella on top.

Operation

Slide the foot under the body while maintaining a gentle vertical force. The foot path should approximate a straight line (if it does not, adjust the 'muscle' lengths). Note how the different muscle elements become taught according to the leg position. Also note that a vertical force can be supported at each position; and how this relates to the moments being generated (/opposed) by the muscles. Simplification: the principles are clear given only two 'muscles'. Extension: given the same origins and insertions, can other horizontal near-straight-lines be achieved with a more flexed leg given different muscle lengths?

Journal of Experimental Biology • Supplementary information

Further files

Code for linkage figures and animations (in LabVIEW 2012, National Instruments, Austin, Texas, USA) at: https://doi.org/10.6084/m9.figshare.16998691.v1

Code_Roberts. Code for Roberts' linkage geometry.

Code_4_bar. Code for forelimb serial 4-bar linkage geometry.

Code_6_bar. Code for hindlimb serial 6-bar linkage geometry.

Scapular and serratus as modified Roberts' linkage

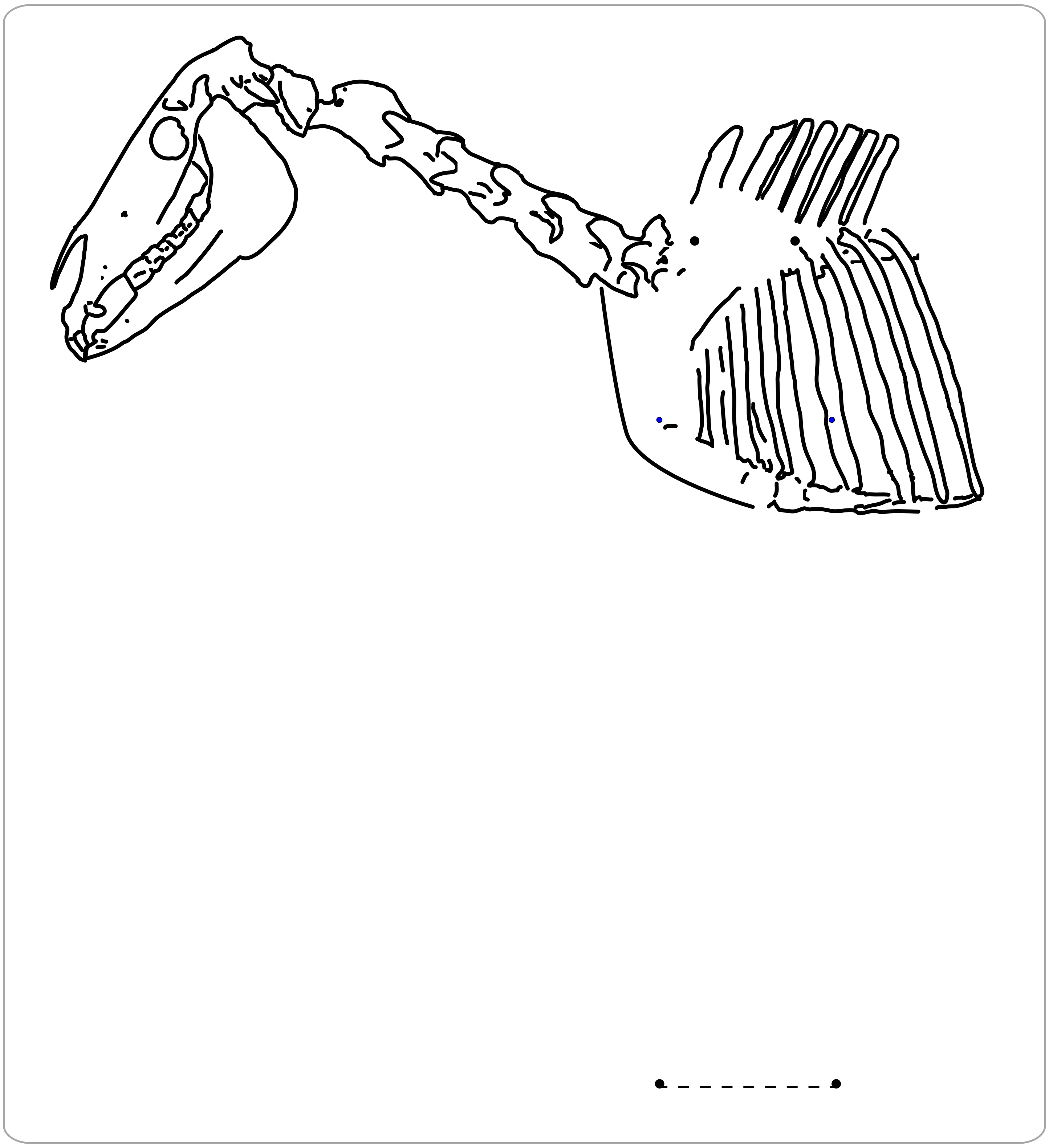

Serratus length: 33.7mm

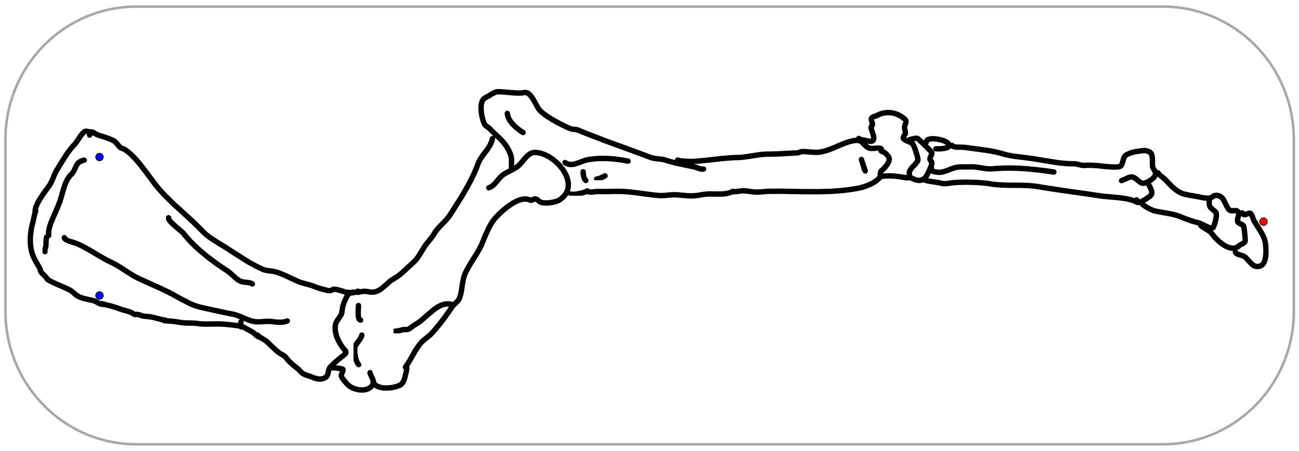

Roberts' forelimb, horse, part and base/body

Journal of Experimental Biology • Supplementary information

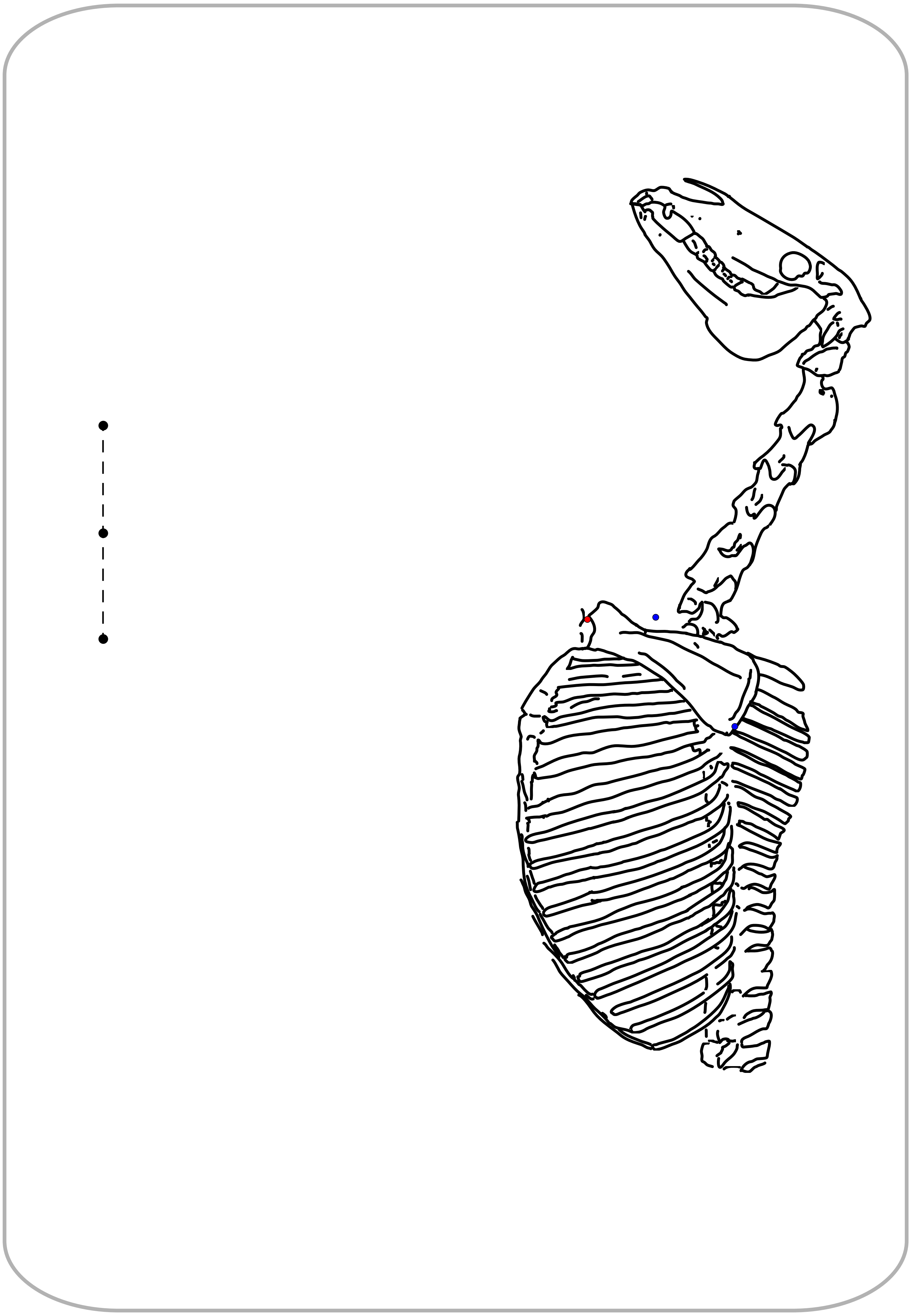

4-bar forelimb, horse, body/base

Journal of Experimental Biology • Supplementary information

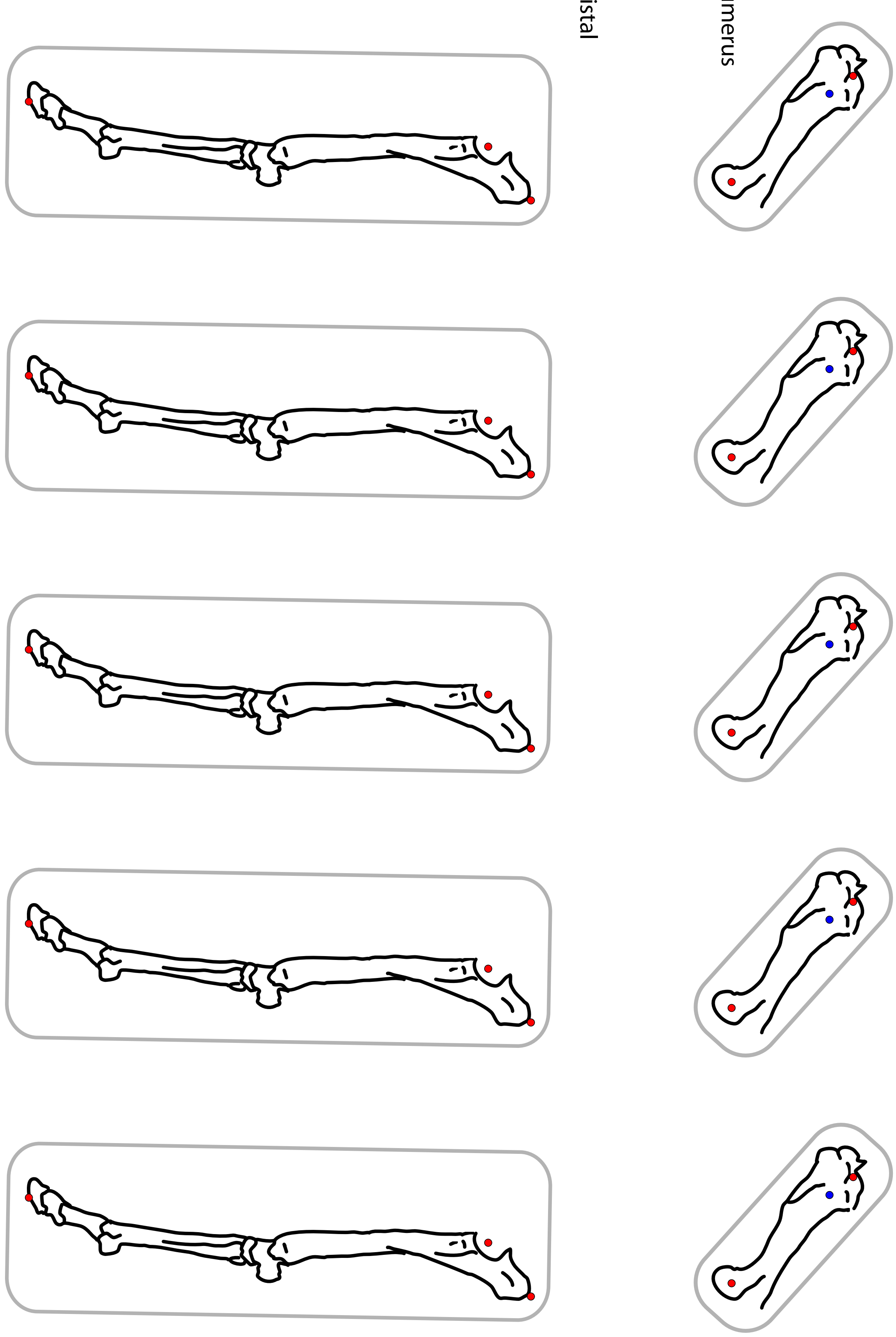
Humerus
Distal
4-bar forelimb, horse, parts

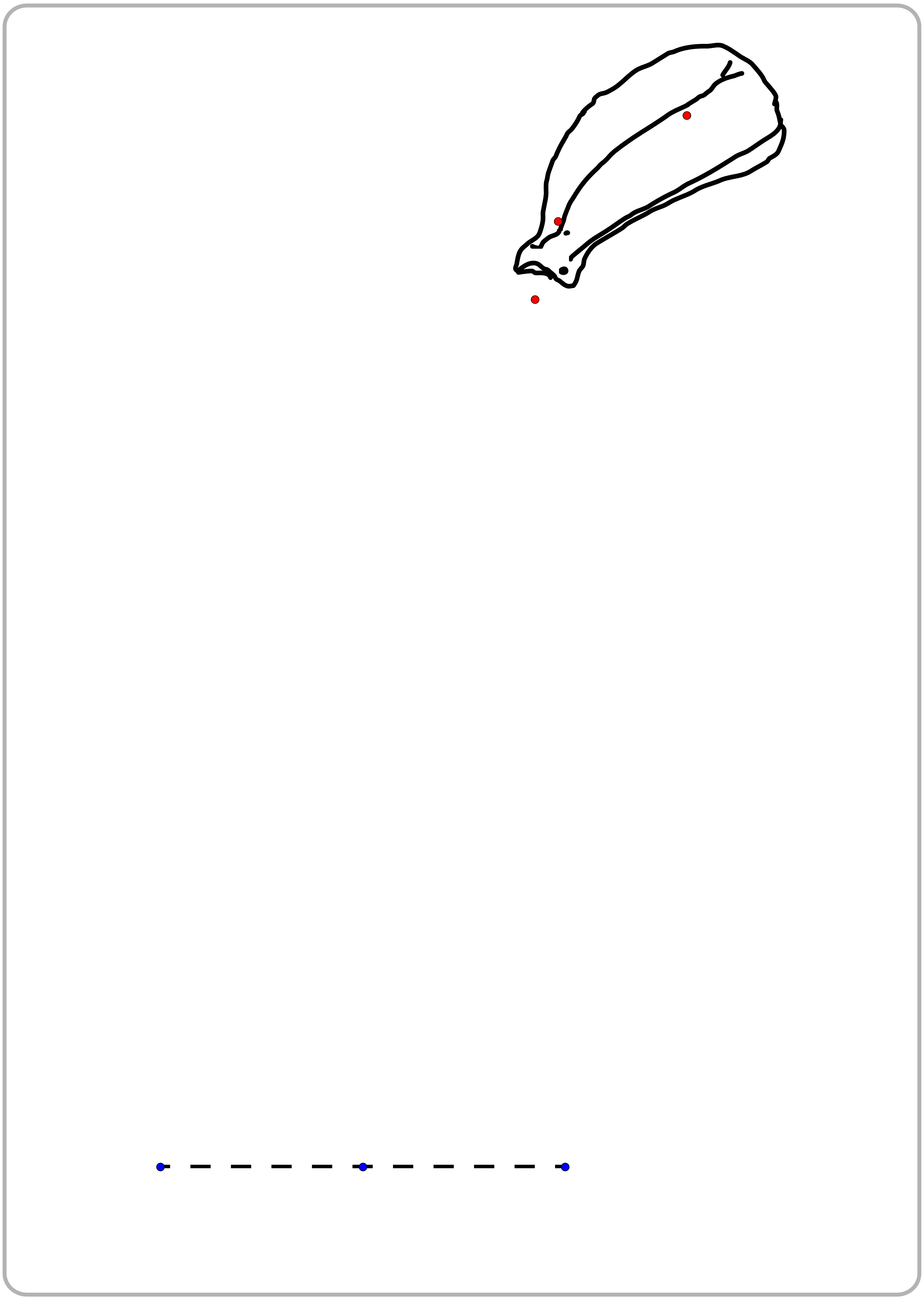

4-bar forelimb, dog, body/base

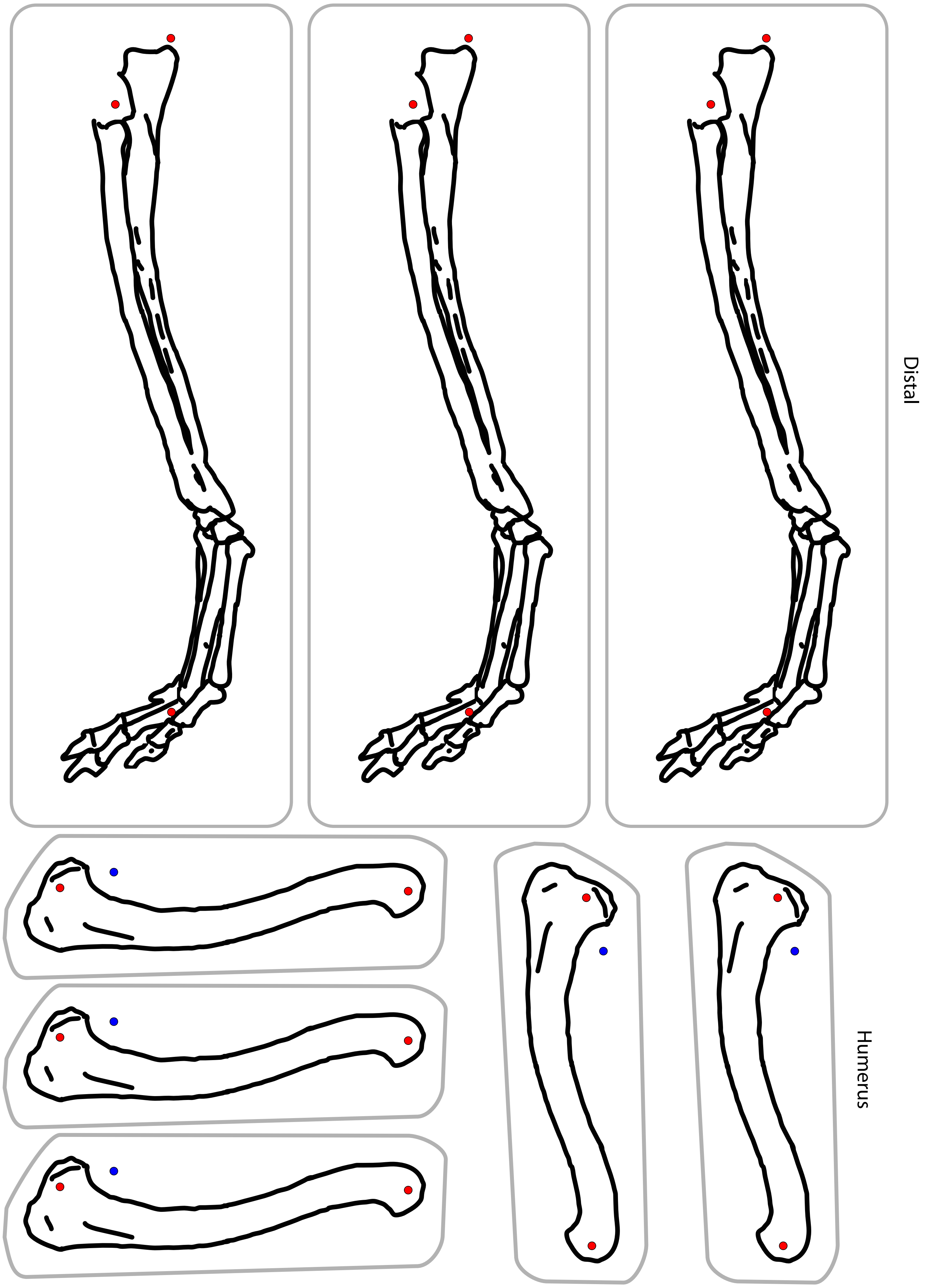

4-bar forelimb, dog, parts

Journal of Experimental Biology • Supplementary information

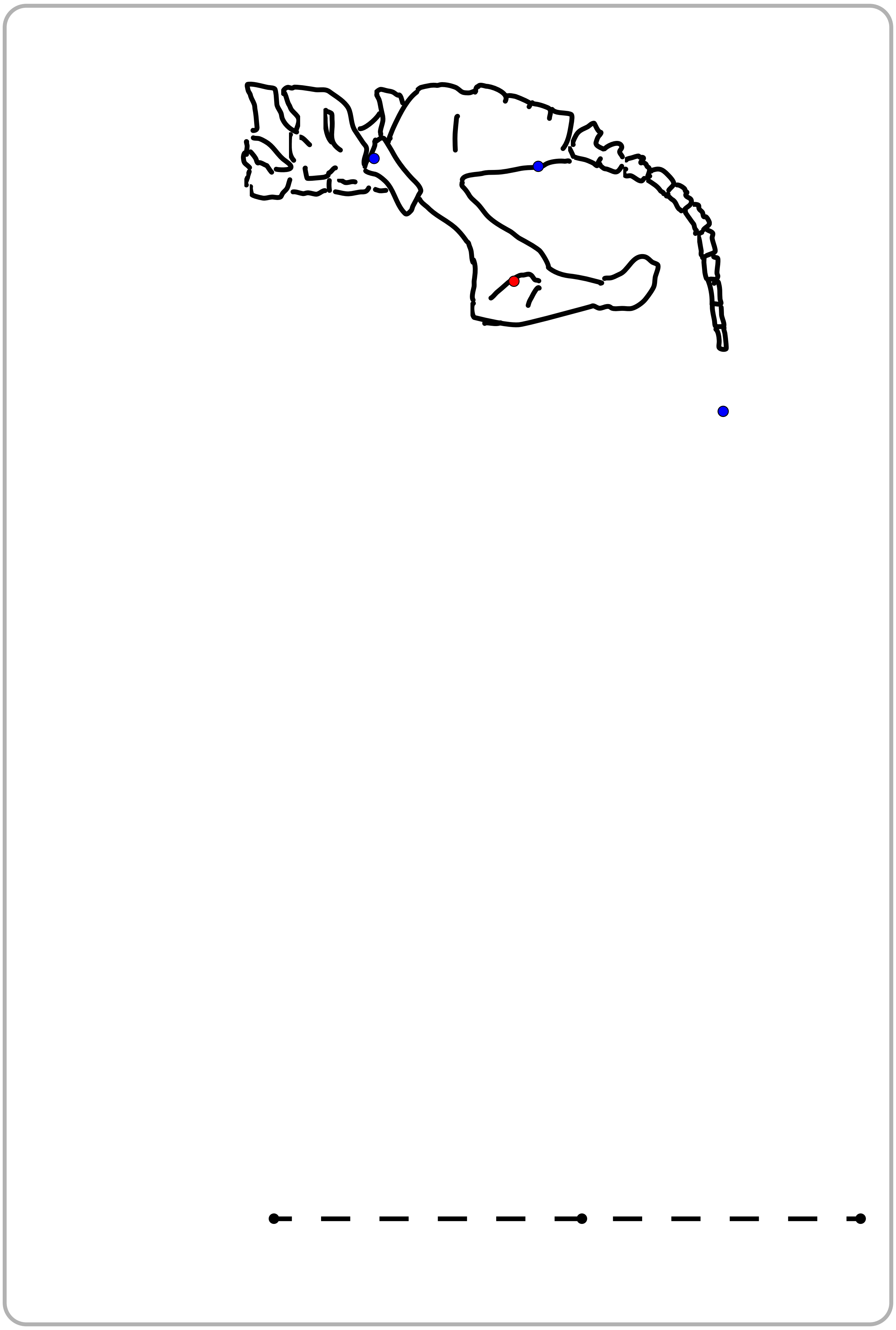

6-bar hindlimb, horse, body/base

Journal of Experimental Biology • Supplementary information

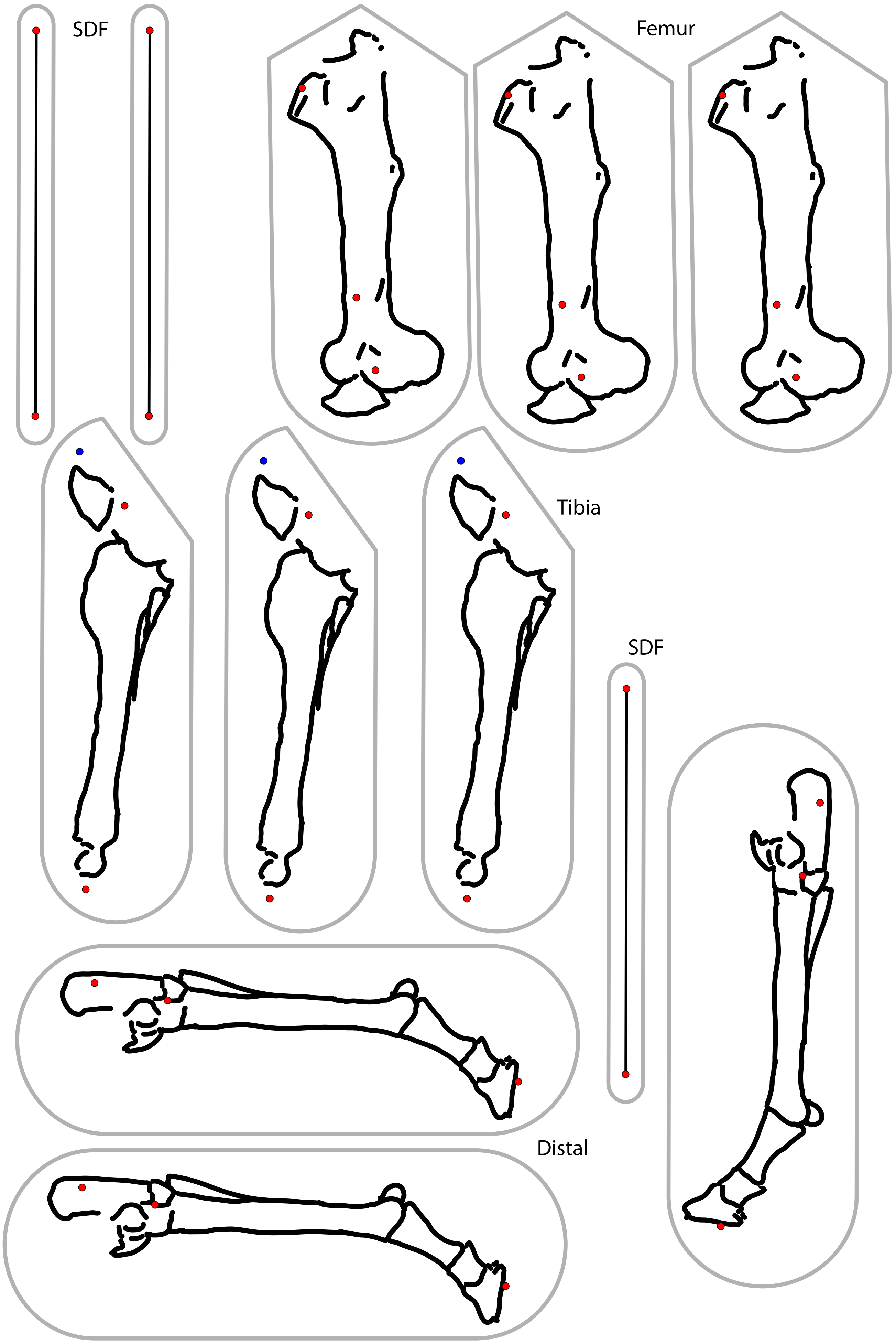

6-bar hindlimb, horse, parts

Journal of Experimental Biology • Supplementary information

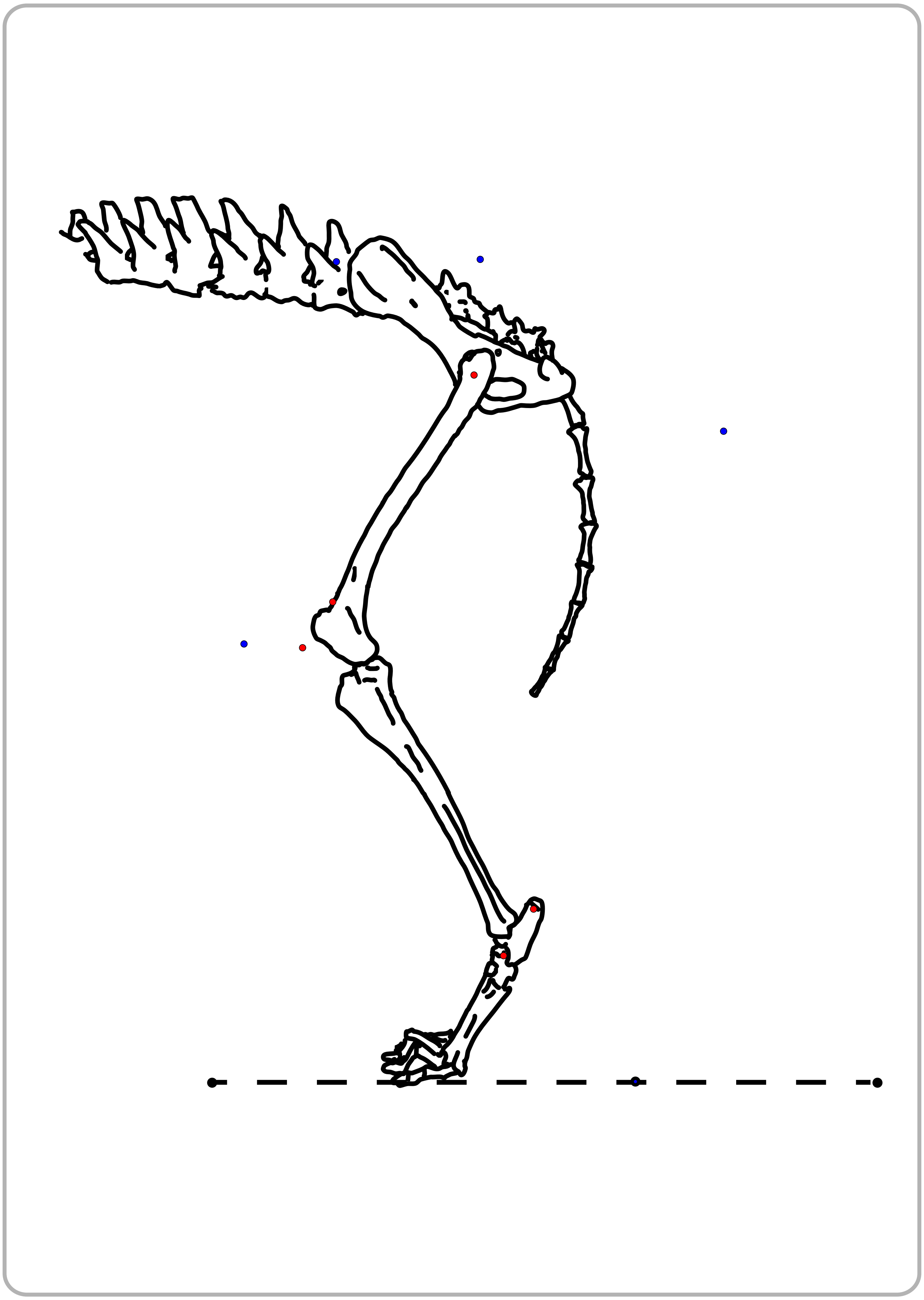

6-bar hindlimb, dog, base/body

Journal of Experimental Biology • Supplementary information

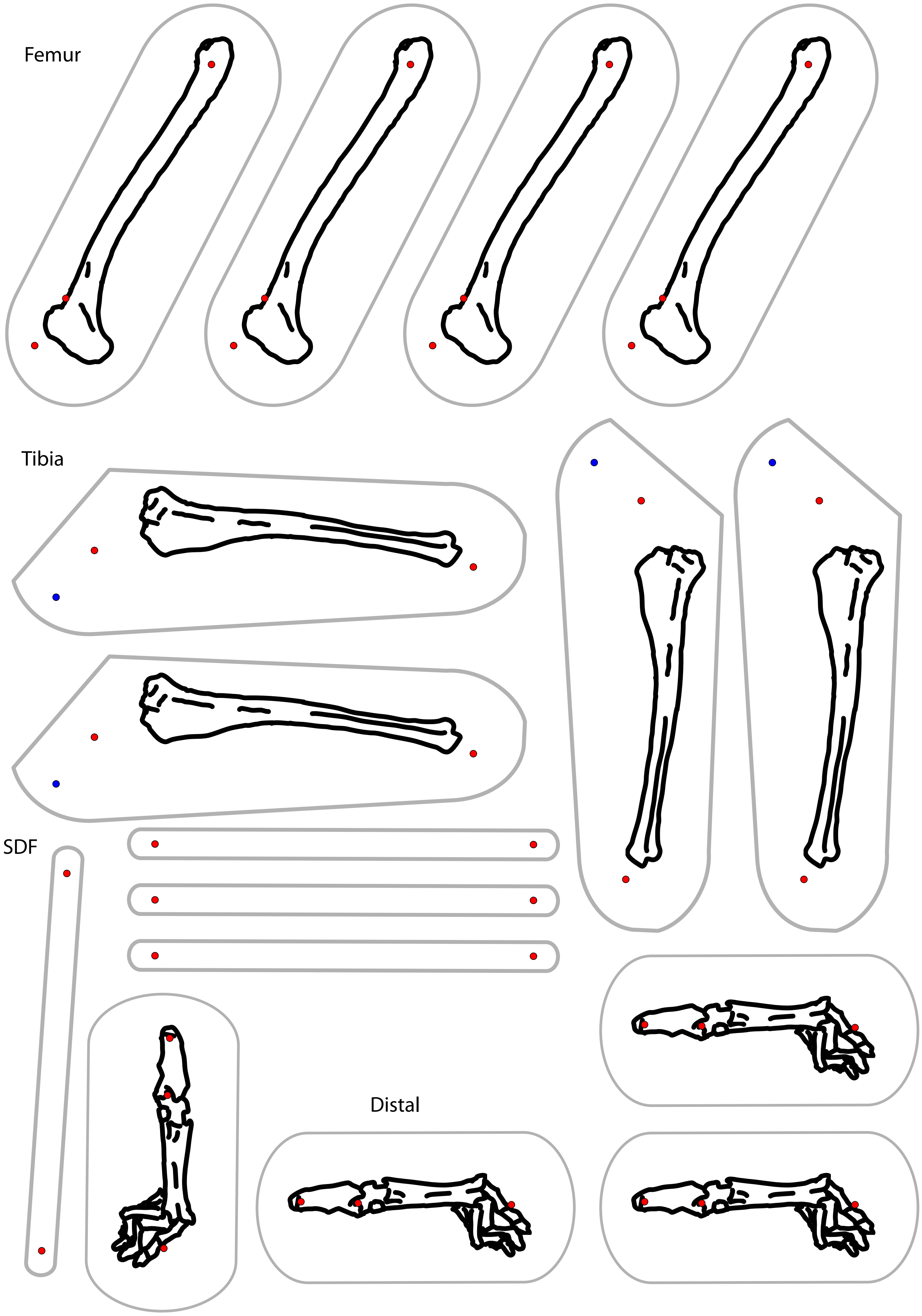

6-bar hindlimb, dog, parts

Journal of Experimental Biology • Supplementary information

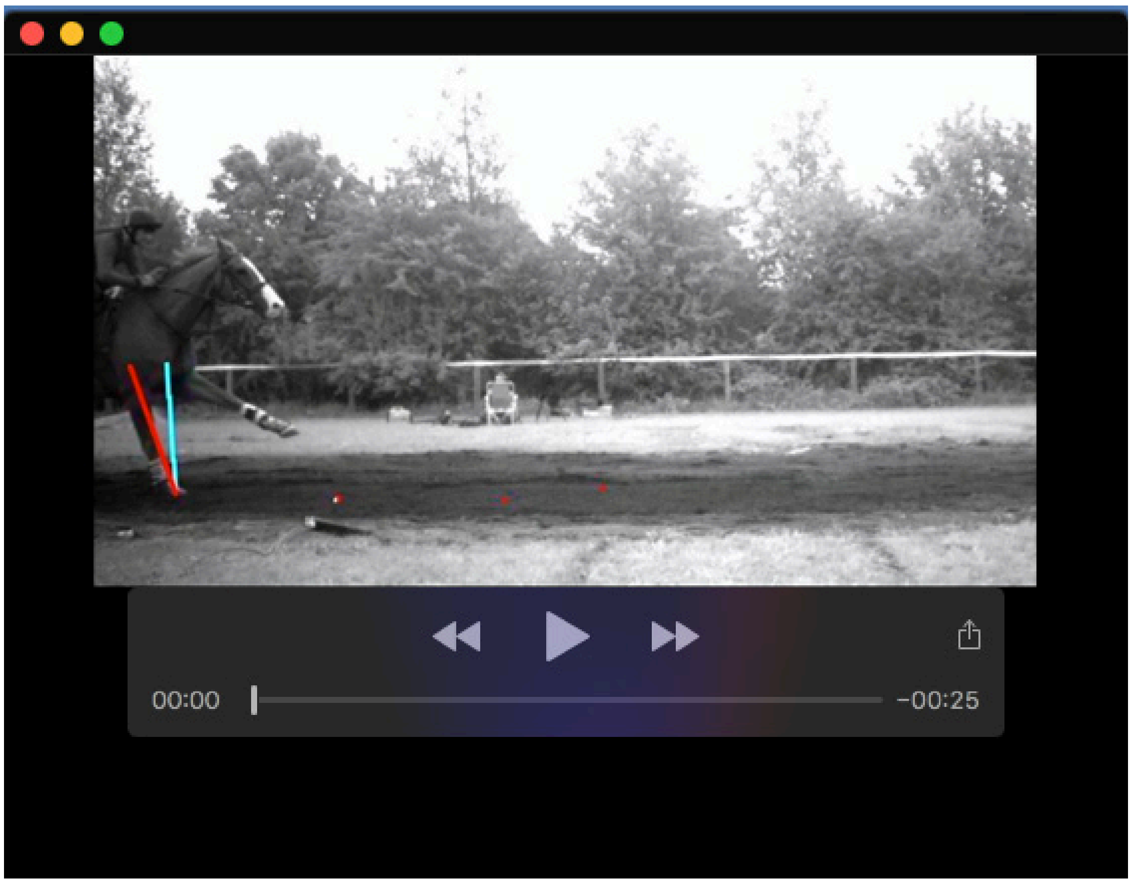

Movie 1. Force vectors of a galloping horse from Self Davies et al., 2019. Measured forces (blue) are closer to vertical than in-line with the legs; they do not project through hips and shoulders (red: empirical vertical forces re-aligned through approximate shoulder and joint centres). Measured forces require 40- 50% of the horizontal work that would be required from limb-axial forces.

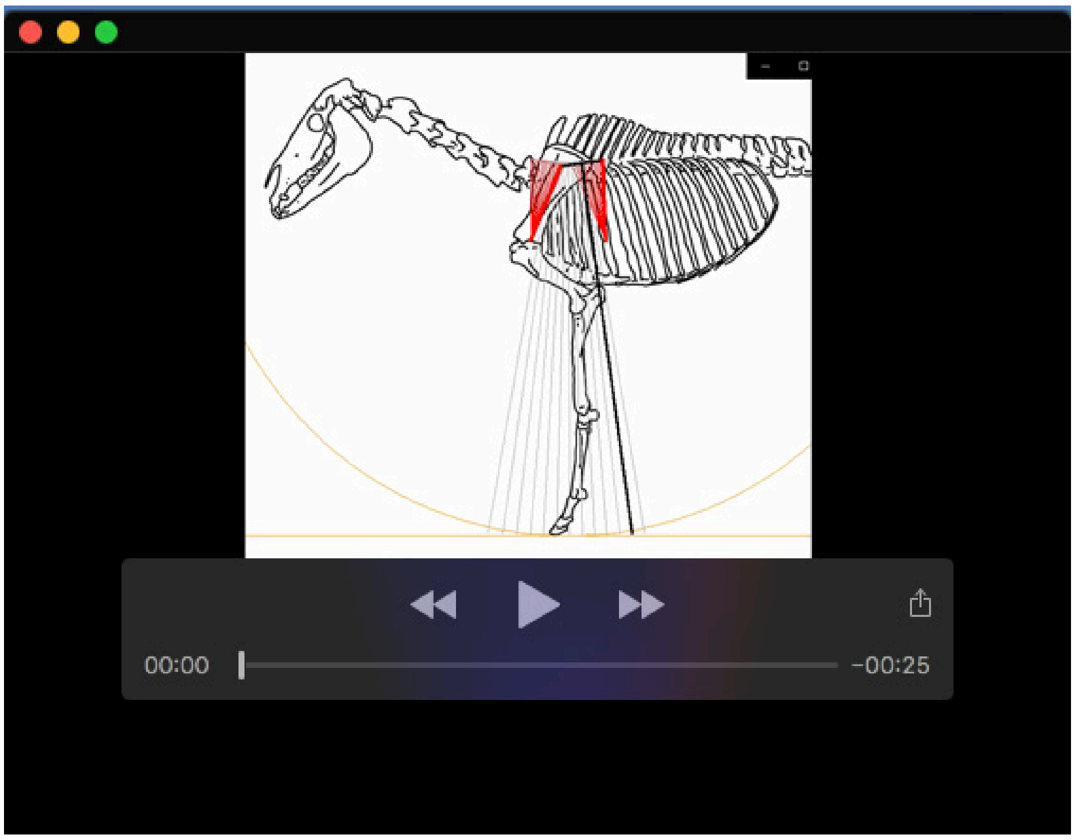

Movie 2. Animation of the horse forelimb modelled as a modified Roberts' straight-line mechanism. See Figure 2.

Journal of Experimental Biology • Supplementary information

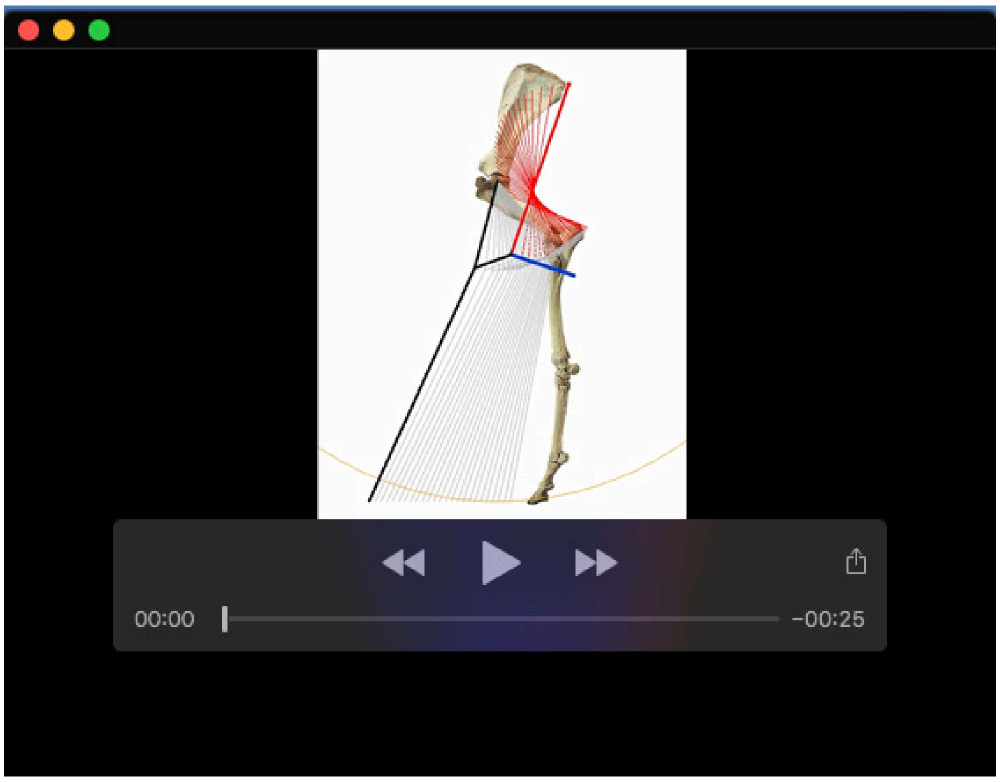

Movie 3. Animation of a horse, then dog, forelimb with horizontal straight-line motion of the foot under the shoulder, vertical limb forces supported by a series of isometric tension elements (red lines) that broadly match the triceps brachii and result in a series of 4-bar linkages. See Figure 3.

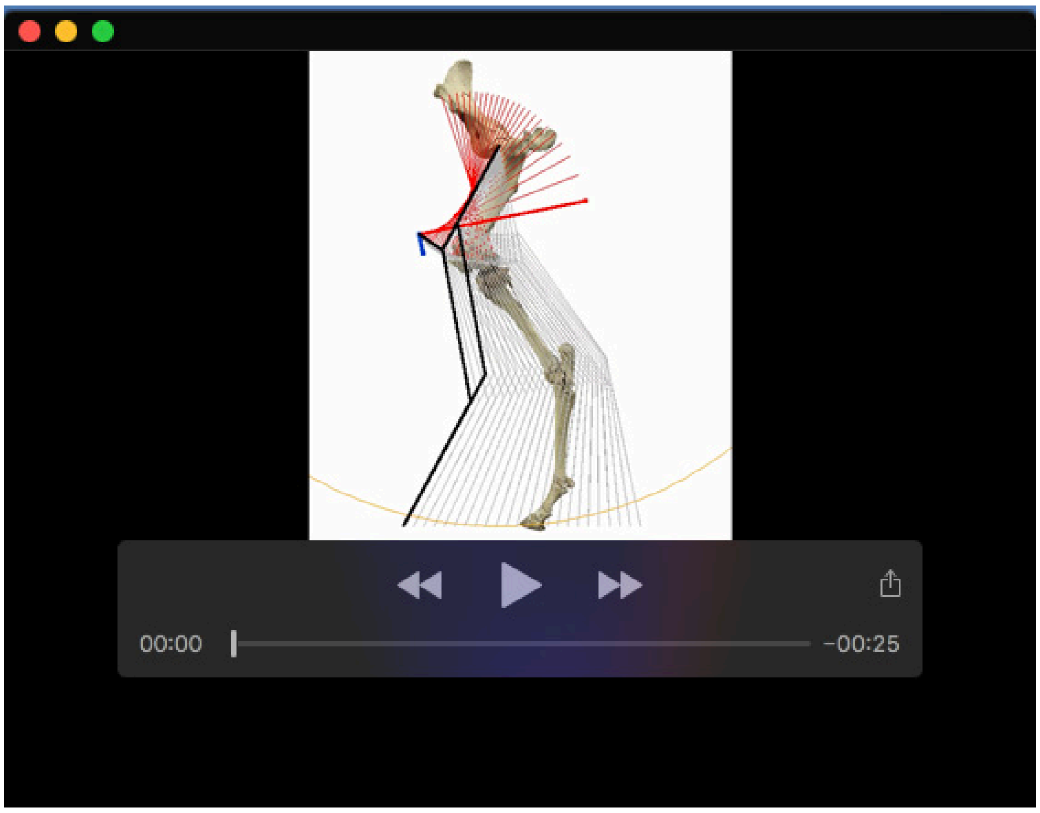

Movie 4. Animation of a horse, then dog, hindlimb with horizontal straight-line motion of the foot under the hip and vertical limb forces supported by a series of isometric tension elements (red lines) that broadly match the cranial femoris (early stance), rectus femoris (midstance) and tensor fascia latae (late stance) and result in a series of Watt's 6-bar linkages. See Figure 5. Note in early stance the cross-over link of the proximal 4-bar linkage formed by the biceps femoris couples limb retraction with limb shortening; and the uncrossed link of the tensor fascia latae, loaded in late stance, couples limb retraction with limb extension.

Journal of Experimental Biology • Supplementary information
